# The Role of Human Coronaviruses in Children Hospitalized for Acute Bronchiolitis, Acute Gastroenteritis, and Febrile Seizures: A 2-Year Prospective Study

**DOI:** 10.1371/journal.pone.0155555

**Published:** 2016-05-12

**Authors:** Monika Jevšnik, Andrej Steyer, Marko Pokorn, Tatjana Mrvič, Štefan Grosek, Franc Strle, Lara Lusa, Miroslav Petrovec

**Affiliations:** 1 Institute of Microbiology and Immunology, Faculty of Medicine, University of Ljubljana, Zaloška 4, 1000, Ljubljana, Slovenia; 2 Department of Infectious Diseases, University Medical Centre Ljubljana, Japljeva 2, 1525, Ljubljana, Slovenia; 3 Department of Pediatric Surgery and Intensive Care, University Medical Centre Ljubljana, and Faculty of Medicine, University of Ljubljana, Bohoričeva 20, 1000, Ljubljana, Slovenia; 4 Institute of Biostatistics and Medical Informatics, Faculty of Medicine, Vrazov trg 2, 1104, Ljubljana, Slovenia; Kliniken der Stadt Köln gGmbH, GERMANY

## Abstract

Human coronaviruses (HCoVs) are associated with a variety of clinical presentations in children, but their role in disease remains uncertain. The objective of our prospective study was to investigate HCoVs associations with various clinical presentations in hospitalized children up to 6 years of age. Children hospitalized with acute bronchiolitis (AB), acute gastroenteritis (AGE), or febrile seizures (FS), and children admitted for elective surgical procedures (healthy controls) were included in the study. In patients with AB, AGE, and FS, a nasopharyngeal (NP) swab and blood sample were obtained upon admission and the follow-up visit 14 days later, whereas in children with AGE a stool sample was also acquired upon admission; in healthy controls a NP swab and stool sample were taken upon admission. Amplification of polymerase 1b gene was used to detect HCoVs in the specimens. HCoVs-positive specimens were also examined for the presence of several other viruses. HCoVs were most often detected in children with FS (19/192, 9.9%, 95% CI: 6–15%), followed by children with AGE (19/218, 8.7%, 95% CI: 5.3–13.3%) and AB (20/308, 6.5%, 95% CI: 4.0–9.8%). The presence of other viruses was a common finding, most frequent in the group of children with AB (19/20, 95%, 95% CI: 75.1–99.8%), followed by FS (10/19, 52.6%, 95% CI: 28.9–75.6%) and AGE (7/19, 36.8%, 95% CI: 16.3–61.6%). In healthy control children HCoVs were detected in 3/156 (1.9%, 95% CI: 0.4–5.5%) NP swabs and 1/150 (0.7%, 95% CI: 0.02–3.3%) stool samples. It seems that an etiological role of HCoVs is most likely in children with FS, considering that they had a higher proportion of positive HCoVs results than patients with AB and those with AGE, and had the highest viral load; however, the co-detection of other viruses was 52.6%.

***Trial Registration***: ClinicalTrials.gov NCT00987519

## Introduction

Human coronaviruses (HCoVs) consist of four species (HKU1, NL63, 229E, and OC43) and are associated with a wide variety of clinical presentations. In otherwise healthy children, HCoVs typically cause mild upper respiratory tract infections, whereas in premature infants and children with chronic underlying diseases they may cause severe lower respiratory tract infections such as pneumonia and bronchiolitis [1–4]. However, the role of HCoVs in severe disease is unclear because they are frequently co-detected with other respiratory viruses, most often with respiratory syncytial virus (RSV) [4, 5]. Among four species of HCoVs, NL63 was found to be more frequently associated with croup than the other three species [6–8], whereas NL63 and HKU1 were associated with bronchiolitis and wheezing [6, 8–14]. Previous studies also suggested an association between HCoVs and involvement of the gastrointestinal and central nervous system. Almost half of patients with HCoVs respiratory infections report abdominal pain, emesis, and diarrhea [15–17], and HCoVs were detected in some patients with symptoms/signs limited to the gastrointestinal tract. Infections with 229E and OC43 have been implicated in the development of various chronic neurologic disorders, including multiple sclerosis [18]; OC43 was demonstrated in the cerebrospinal fluid of a child presumed to have acute disseminated encephalomyelitis [19]; and HKU1 has been associated with febrile seizures [12].

The aim of the study was to determine and compare the frequency of HCoVs in children with acute bronchiolitis (AB), acute gastroenteritis (AGE), and/or febrile seizures (FS), and to assess their etiological role in each individual syndrome.

## Materials and Methods

### Study population

The study presented here was a part of a prospective study on viral respiratory and gastrointestinal infections in children under 6 years of age. The study protocol was approved by the National Medical Ethics Committee of the Republic of Slovenia (No. 87/08/09) and was registered at the ClinicalTrials.gov registry (reg. NCT00987519). Written informed consent was obtained from the parents of all participating patients and control subjects. The principles of the Helsinki Declaration, the Oviedo Convention on Human Rights and Biomedicine, and the Slovene Code of Medical Deontology were strictly followed in this study.

Children under 6 years of age, admitted to the Department of Infectious Diseases, University Medical Centre Ljubljana, from 10^th^ October 2009 to 24^th^ September 2011 (for participant) and from 23^rd^ October 2009 to 7^th^ October 2011 (for follow-up) with a diagnosis of AB (defined as the presence of nasal discharge, cough, wheezing, and/or crackles on lung auscultation), AGE (defined as passage of at least three or more loose or liquid stools in 24 hours requiring parenteral rehydration), and/or FS (defined as a cerebral paroxysm accompanied by fever without signs of central nervous system infection) were eligible for the study. Children with more than one clinical manifestation (i.e., AB and AGE) were classified under the diagnosis that had been the main reason for hospital admission. Nasopharyngeal (NP) swabs and blood samples were obtained upon admission and at a follow-up visit 14 days after initial sampling. In children with AGE a stool sample was also acquired upon admission to hospital. In an individual patient only one sample from the particular source was taken at each of the two time points. Findings in patients with AGE have been reported previously [12, 20].

The healthy control group comprised children under 6 years old admitted to the Department of Pediatric Surgery and Intensive Care for elective surgical procedures (mainly for inguinal hernia, testicular retention, and hydrocele testis) during the same time period as the study subjects. In addition to the general rules requiring that only children without infections within the last four weeks prior to surgery be admitted for elective surgical procedures (and that the procedure is postponed in children with symptoms/signs of infection found at examination prior to surgery), this study also obtained additional specific information on the presence of signs and symptoms compatible with gastrointestinal and/or respiratory infection within the last 14 days. The NP swab and stool specimen were obtained from the control group participants upon admission; in order to minimize discomfort, nasopharyngeal sampling was performed after the children underwent general anesthesia.

In all participants an NP swab acquired at the initial examination was mandatory for the inclusion in the study; in patients with AGE a stool sample was also needed.

### Sample preparation and nucleic acid extraction

NP swabs were collected using flocked-tip swabs and transported to the laboratory of the Institute of Microbiology and Immunology, Faculty of Medicine, University of Ljubljana in a Copan universal transport medium (UTM-RT) system (Copan Italia, Brescia, Italy).

Stool samples were diluted in sterile phosphate-buffered saline (PBS) to a 10% stool suspension. Aliquots of 180 μL of MagNA Pure Bacteria Lyses Buffer (Roche Applied Science, Mannheim, Germany) and 20 μL of proteinase K (Qiagen, Hilden, Germany) were added to 190 μL of stool suspension.

Before the extraction procedure, 5 μL of Equine herpesvirus 1 and 5 μL of Equine arteritis virus isolates were added to all samples for external DNA and RNA control. Specific target sequences of these viruses were subsequently amplified in separate real-time reverse transcription polymerase chain reactions (RT-PCRs) as an internal control to ensure that negative results were not caused by poor nucleic acid extraction or inhibition of the RT-PCR assay [21, 22].

The initial volume used for extracting total nucleic acids was 190 μL of vigorously vortexed NP swab medium, 400 μL of stool suspension, and 190 μL of whole blood samples. Nucleic acids were extracted using total nucleic acid isolation kits on a MagNa Pure Compact instrument (Roche Applied Science, Mannheim, Germany), according to the manufacturer’s instructions.

### Detection methods

Coronaviruses including HKU1, NL63, 229E, and OC43 were tested in NP swabs, stool samples, and whole blood samples (blood was examined for the presence of HCoVs only in children with positive NP swabs and/or stool results) by molecular methods using primers and probes as described by Kuypers et al. [4]. For amplification of 85–100 bp fragments of the coronaviruses’ polymerase 1b gene, a one-step real-time RT-PCR assay was used in a StepOne Real-Time PCR system (Applied Biosystems, Carlsbad, CA). Briefly, 5 μL of total nucleic acid was added to 15 μL of reaction mixture including 2 × Reaction Mix, SuperScript^®^ III RT/Platinum^®^ Taq Mix (Invitrogen, Carlsbad, CA) with an additional 6 mM M_g_SO_4_. The cycling conditions were universal for all respiratory viruses tested: 20 min at 50°C, 2 min at 95°C, and 45 cycles of 15 s at 95°C and 45 s at 60°C.

All NP swabs and stool samples in which HCoVs were established were also tested for the presence of several other viruses. In NP swabs respiratory syncytial virus (RSV), influenza viruses A and B (Inf A-B), parainfluenza viruses 1–3 (PIV 1–3), human metapneumovirus (hMPV), human bocavirus (HBoVs), adenovirus (AdV) and human rhinovirus (hRV) were searched for by real-time RT-PCR [23–29]. Stool samples from patients with AGE were tested for the presence of noroviruses of genogroups I and II, human astroviruses, HBoV and AdV using molecular methods as described previously [27, 28, 30, 31], whereas group A rotavirus and adenovirus type 40/41 were detected by antigen-ELISA Premier Rotaclone and Premier Adenoclone (Meridian Bioscience, Cincinnati, OH).

### Sequence Analysis

Sequencing was performed to confirm the specificity of the RT-PCR assay using primers pairs for group 1 subtypes (HCoV-F2 and HCoV-R2) and for group 2 subtypes (HCoV-F1 and HCoV-R1) to amplify 823 bp and 912 bp fragments of the polymerase 1b gene of HCoVs, respectively, using Promega PCR Master Mix (Promega, Madison, WI) (Table 1). PCR products were purified and sequenced subsequently using BigDye terminator chemistry on an ABI PRISM 310 genetic analyzer (Applied Biosystems).

**Table 1:** Selected primers for the sequence detection of polymerase 1b gene.

Primer	Sequence, 5′→ 3′	HCoV subtype	Nucleotide position^a^
HCoV-F1	TGAGTGATGATGGKGTTGT	2	15576–15594
HCoV-R1	GTTGCCTTTTGMGTTTCTG	2	16521–16503
HCoV-F2	GRGTTGAGTGTTATAGTGG	1	16151–16167
HCoV-R2	GCATGASTTGGTGGTAAA	1	16989–16972

^a^ Primers positions are given according to their position on the species OC43 and NL63 whole genome sequence, GenBank accession numbers KF923925.1 and KF530114.1.

### Statistical Analysis

Numerical data were summarized with medians (interquartile range, IQR), categorical data with frequencies, and percentages. 95% confidence intervals (CI) for percentages were based on exact binomial distributions. Univariate comparisons were based on the Mann-Whitney test for numerical variables and on the chi-squared test for categorical variables.

Odds ratios (OR) with a 95% CI were used to quantify the strength of association between categorical variables. Multivariable logistic regression was used to adjust the analyses for additional covariates (such as age and sex).

McNemar’s chi-squared test was used to assess the association between categorical variables measured in the same patients (at baseline and at the follow-up visit, in NP swabs and stool samples).

The association between HCoVs positivity and seasonality was assessed using logistic regression, in which HCoVs positivity was the response variable and the calendar day of admission to hospital (number of days from the first of January) was the covariate, modelled using restricted cubic splines (RCS) with four knots. The aim of this analysis was to display graphically the estimated association was between HCoV positivity and calendar day. Using RCS we avoided the a priori assumption of linearity (on the logit scale) between the calendar day and HCoV positivity.

The association between HCoVs positivity at the follow-up visit and at baseline was estimated using a logistic regression model with HCoVs positivity at follow-up as the outcome variable and HCoVs positivity at baseline as covariate; the analysis was also adjusted for the children’s sex and age. A similar multivariable logistic regression model was used to assess the association between HCoVs positivity at follow-up and baseline positivity for other viruses (RSV, hMPV, Inf, HBoV, hRV, PIV, Ad, and HCoVs).

All analyses were performed using R statistical language [32] using the functions included in the base and in the stats packages. For restricted cubic splines we used the rcs() function from the rms package.

## Results

### Patients and controls

During a 2-year period (from October 2009 to September 2011), 6164 children under 6 years of age were admitted to the Department of Infectious Diseases, including 814 children with a diagnosis of AB, 942 children with AGE, and 278 children with FS. Of 2034 patients with AB, AGE or FS, 718 (34.3%) were included in the study. The others were not enrolled because their parents did not consent to their inclusion in the study (1243 children) or because they did not pass stool during their hospital stay even though they were admitted for AGE (73 children).

Of 809 children admitted for elective surgery and whose parents were asked to give consent for them to take part in the study, 156 (19.2%) were enrolled. The main reason for non-enrollment was that two other studies whose rules precluded inclusion in our study were being conducted simultaneously.

The median age of the study participants was 17.6 months (IQR: 10.5–25.8), with a female:male ratio of 1:1.6. Children with acute infections were younger than controls (median age 16.7 months, IQR: 9.6–23.4 months versus median age 25.8 months, IQR: 14.6–45.8 months, P<0.001) and were less often boys (404/718 (56.3%) among cases and 132/156 (84.6%) among controls, P<0.001).

Of 718 children with infections, 308 (42.9%) had AB, 192 (26.7%) FS (132 (68.7%) simple, 60 (31.3%) complex), and 218 (30.4%) AGE. Some children had more than one clinical syndrome: 21 had AB and AGE, 8 had AB and FS, 21 had FS and AGE, and 1 child had clinical indications of all three syndromes. Children with AB were younger compared to the other two groups (AB: median age 11.7 months, IQR: 5–20; FS: median age 18.3 months, IQR 14.7–27.4; AGE: median age 19.5, IQR 13.8–28.3).

### Samples

718 NP swabs and 677 blood samples were obtained from 718 children with AB, FS, and/or AGE at enrollment; in addition, a stool sample was acquired from 218/218 children with AGE. 156 NP swabs and 150 stool samples were acquired from the 156 children comprising the healthy control group.

At follow-up examination 14 days after initial testing, NP swabs were available for 537/718 (74.8%) children, including 237/308 (77%) patients having had AB, 143/192 (74.5%) patients that had suffered from FS, and 157/218 (72%) patients that had been hospitalized for AGE. In 514/537 (96%) children from whom NP swabs were acquired blood samples were also obtained. Children with follow-up data were younger than those without follow-up (2.5 months of average difference), but the two groups were comparable according to sex.

### HCoVs detection

The presence of HCoVs upon admission was established in 58/718 (8.1%, 95% CI: 6.2–10.3%) NP swabs obtained from patients with AB, FS, or AGE, and in 6/218 (2.7%, 95% CI: 1.0–5.9%) stool samples of children with AGE. The corresponding findings in the control group were 3/156 (1.9%, 95% CI 0.4–5.5%) and 1/150 (0.7%, 95% CI 0–3.6%) for NP swabs and stool specimens, respectively. All children included in the control group were asymptomatic on the day of sampling, but a detailed history revealed that in three cases parents recalled symptoms indicating respiratory or gastrointestinal infection within 14 days before sampling. HCoVs in NP swabs were detected more often in children with AB, FS, and AGE than in control group children (8.1% versus 1.9%, OR = 4.5, 95% CI: 1.4–14.5%, P = 0.01) and the magnitude of the association also remained similar when the analysis was adjusted for patients’ age and sex (OR = 4.5, 95% CI: 1.3–15.2%, P = 0.02). The same was valid also for individual syndromes: FS (9.9% versus 1.9%, OR = 5.60, 95% CI: 1.63–19.29, P = 0.006), AGE (8.7% versus 1.9%, OR = 4.87, 95% CI: 1.42–16.75, P = 0.01), and AB (6.5% versus 1.9%, OR = 3.54, 95% CI: 1.04–12.11%, P = 0.04). The virus was not found in the blood of any patients with positive HCoV NP swab or stool sample results.

Patients with FS (9.9%, 95% CI: 6–15) and AGE (8.7%, 95% CI: 5.3–13.2) had a higher probability of having a positive HCoVs result compared to those with AB (6.5%, 95% CI: 4.0–9.8) but the association was not statistically significant. Of 19 HCoVs positive children with FS 11 (57.9%, 95% CI: 33.5–79.8) had simple and 8 (42.1%) had complex febrile seizures (5 of these 8 children required anticonvulsant treatment), while in HCoVs negative children with FS 121/173 (69.9%, 95% CI: 62.5–76.7) had simple and 52/173 (30.1%) had complex FS (31 of these 52 required anticonvulsant treatment). In children with AGE, HCoVs were more often detected in NP swabs than in stool samples (19/218, 8.7%, 95% CI 5.3–13.2% in NP swabs versus 6/218, 2.7%, 95% CI 1.0–5.9% in stool samples; P<0.001).

Among patients with established HCoVs in NP swabs, the estimated viral load was the highest in children with FS (median cycle threshold (Ct) value 27.8, IQR 27.45–36.02), followed by AGE (median 29.5, IQR 24.40–35.50) and AB (median 33.3, IQR 27.45–36.02). Ct values in the three children with positive HCoVs in NP swabs belonging to the control group were high (31.7, 33.4, and 39.3, respectively).

**Fig 1:**
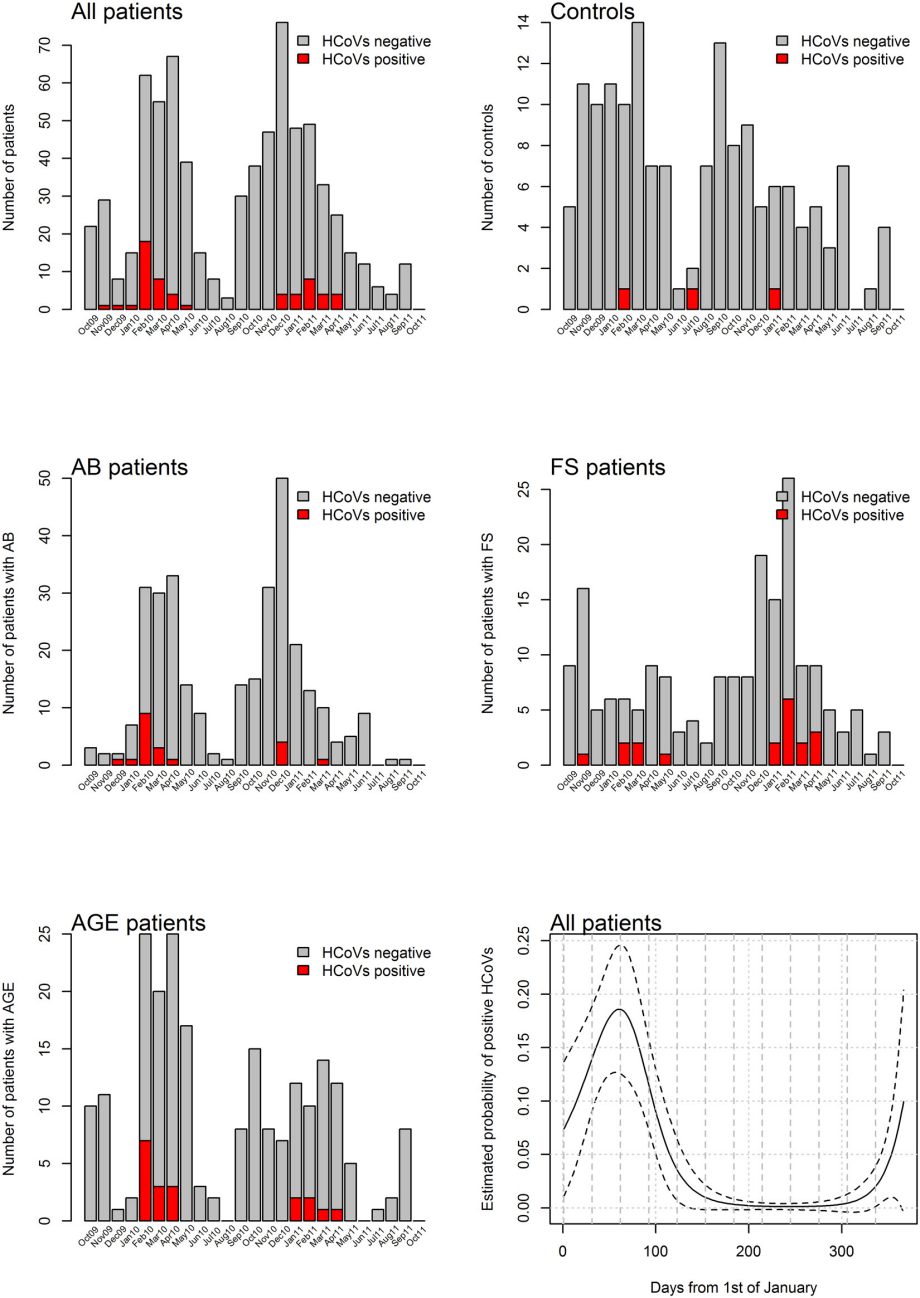
Seasonal distribution of HCoVs-positive samples. Human coronaviruses (HCoVs), acute bronchiolitis (AB), febrile seizures (FS), and acute gastroenteritis (AGE).

Most HCoVs-positive cases were detected in winter (41/58, 70.7%) followed by spring (14/58, 24.1%), whereas only 3/58 (5.1%) cases were detected in autumn and none in summer. The association between HCoVs positivity and seasonality (calendar day of admission to hospital) was statistically significant (P<0.001). More HCoVs-positive cases were detected in winter 2009/2010 (34/58, 58.6%) than in winter 2010/2011 (24/58, 41.4%). In both years the highest probability of HCoVs-positive results was estimated in February and March (Fig 1).

Of 58 HCoVs detected in NP swabs obtained upon admission from patients with infection, 23 (39.6%) belonged to OC43, 19 (32.7%) to HKU1, 8 to NL63 (13.8%), and 5 (8.6%) to 229E, whereas 3 HCoV-positive NP samples remained untyped because of their low viral load (high Ct value). Three NP specimens contained two HCoVs in different combinations, including NL63/OC43, NL63/HKU1, and 229E/HKU1. The primary species was determined according to the Ct value (the species with the lower Ct value was interpreted as primary). OC43 was detected most frequently in children with FS (12/19, 63.1%), whereas HKU1 was the most common HCoV among children with AB (9/20, 45%) and AGE (7/19, 36.8%) (Table 2). Of four HCoVs detected in healthy controls one virus was typed as NL63, one as 229E, and two HCoVs remained untyped because of their low viral load.

**Table 2:** Demonstration of HCoVs species in NP swabs obtained from children with acute bronchiolitis (AB), febrile seizures (FS), and acute gastroenteritis (AGE).

		AB	FS	AGE	Total
229E	Total	2/20, 10%	1/19, 5.3%	2/19, 10.5%	5/58, 8.6%
	229E only	0/2	1/1	2/2	3/5
	229E + additional virus(es)	2/2	0/1	0/2	2/5
HKU1	Total	9/20, 45%	3/19, 15.8%	7/19, 36.8%	19/58, 32.7%
	HKU1 only	1/9	0/3	5/7	6/19
	HKU1 + additional virus(es)	8/9	3/3	2/7	13/19
NL63	Total	5/20, 25%	0/19	3/19, 15.8%	8/58, 13.8%
	NL63 only	0/5	0	0/3	0/8
	NL63 + additional virus(es)	5/5	0	3/3	8/8
OC43	Total	4/20, 20%	12/19, 63.1%	7/19, 36.8%	23/58, 39.6%
	OC43 only	0/4	6/12	5/7	11/23
	OC43 + additional virus(es)	4/4	6/12	2/7	12/23
Untyped	Total	0	3	0	3/58, 5.2%
	HCoVs only	0	2/3		2/3
	HCoVs + additional virus(es)	0	1/3		1/3

Untyped, species not determined due to low viral load.

### Presence of additional viruses

In 36 (62.1%) of the 58 HCoVs-positive NP samples other respiratory viruses were detected, most commonly RSV (22/36, 61.1%) (Table 3). NL63 was most often (8/8, 100%) associated with other respiratory viruses, followed by HKU1 (13/19, 68.4%), OC43 (12/23, 52.2%), and 229E (2/5, 40%). 229E was detected as a single viral pathogen only in children with AGE and FS (Table 2).

**Table 3:** Demonstration of respiratory viruses in addition to HCoVs in NP swabs obtained from children with acute bronchiolitis (AB), febrile seizures (FS), and acute gastroenteritis (AGE).

	AB	FS	AGE	Total
HCoV*	20/308 (6.5%)	19/192, 9.9%	19/218, 8.7%	58/718, 8.1%
HCoV only	1/20 (5%)	9/19, 47.4%	12/19, 63.1%	22/58, 37.9%
HCoV + additional virus(es)	19/20 (95%)	10/19, 52.6%	7/19, 36.8%	36/58, 62.1%
HCoV + 1 additional virus	13/19	5/10	3/7	21/36
HCoV + 2 additional viruses	5/19	2/10	3/7	10/36
HCoV + 3 additional viruses	1/17	0	1/7	2/36
HCoV + 4 additional viruses	0	3/10	0	3/36
Additional viruses:				
AdV	4/19, 21%	5/10, 50%	3/7, 42.9%	12
InfA	0	1/10, 10%	1/7, 1.4%	2
InfB	0	2/10, 20%	0	2
PIV 1–3	0	0	1/7, 1.4%	1
RSV	15/19, 78.9%	5/10, 50%	2/7, 28.6%	22
hMPV	4/19, 21%	0	0	4
HBoV	1/19, 5.3%	3/10, 30%	2/7, 28.6%	6
hRV	1/19, 5.3%	2/8, 2%	3/7, 42.8%	6

*Proportion of patients with HCoV demonstrated in NP swab.

AdV, adenovirus; InfA, influenza virus A; InfB, influenza virus B; PIV 1–3, parainfluenza virus 1–3, RSV, respiratory syncytial virus; hMPV, human metapneumovirus; HBoV, human bocavirus; hRV, rhinovirus.

Within HCoVs-positive patients those with AB had a significantly larger probability of having “co-infections” compared to other disease groups (19/20, 95%, 95% CI: 75.1–99.8%; 10/19, 52.6%, 95% CI: 28.9–75.6%; and 7/19, 36.8%, 95% CI: 16.4–57.3% in children with AB, FS, and AGE, respectively; P<0.001). The association remained statistically significant also after adjustment for the children’s age and sex.

HCoVs median Ct values were 27.5 (IQR: 21.8–34.9) in single “infections” and 32 (IQR: 27–35.8) in “co-infections” (P = 0.09).

### Follow-up testing

Of 537 follow-up NP swabs, 35 (6.5%, 95% CI: 4.6–8.9) were positive for HCoVs. The proportion was smaller compared to findings at baseline (58/718; 8.1, 95% CI: 6.2–10.3), but the difference was small and not statistically significant (P = 0.3). 14/35 (40%) HCoVs-positive children were asymptomatic but the others had respiratory and/or gastrointestinal symptoms. Only 10/35 (29%) were HCoVs-positive also at baseline. Patients that were HCoVs-positive at initial sampling were more likely to be HCoVs-positive at the follow-up examination (OR = 5.5 for HCoVs-positivity, 95% CI: 2.4–12.4, P<0.001). The estimated association remained similar and statistically significant also when the analysis was adjusted for age and sex. A multivariable logistic regression model that included RSV, hMPV, Inf, HBoV, hRV, PIV, AdV, and HCoV at baseline as covariates revealed that patients that were HBoV-positive at the initial examination had more likely HCoVs-positive result at follow-up (OR = 4.3, 95% CI: 1.8 to 10.2, P<0.001).

**Fig 2:**
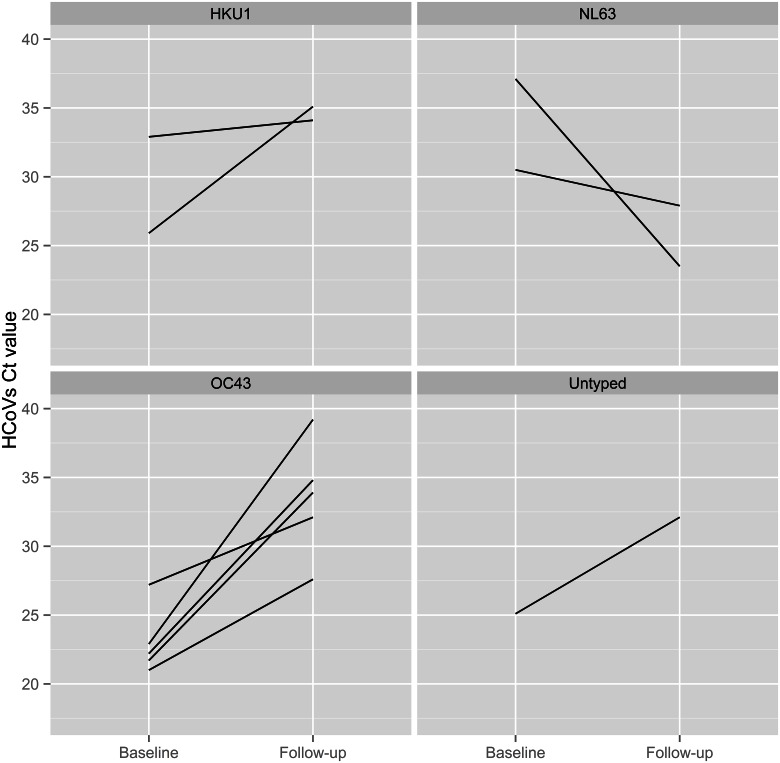
The Ct value measured for HCoV-positive patients at baseline and follow-up examination in nasopharyngeal swabs. HCoV species were not determined due to low viral load (Untyped); HCoV species (229E, HKU1, NL63, OC43).

The Ct value measured for 10 patients that were HCoVs-positive both at baseline and follow-up are displayed graphically (Fig 2). All HCoVs species, except NL63, had higher Ct values at follow-up than at base line. In all nine patients that were positive for HCoVs at the initial and at follow-up sampling visit, and had known HCoV species, the same HCoV species was detected on both occasions.

None of the 35 children positive for HCoVs at follow-up had viruses detected in blood samples taken at the same time.

### Sequencing

In 12 HCoVs the 805-bp-long region of the polymerase 1b gene for coronaviruses from *Alphacoronavirus* and 912-bp-long region for coronaviruses from *Betacoronavirus* were amplified and sequenced. Five of 12 sequences were unique and were deposited in GenBank (acc. no. KF668028-KF668032).

## Discussion

HCoVs have been associated with a wide variety of clinical presentations, but their etiological role has not been fully elucidated and remains uncertain. In this study we determined the occurrence of the viruses by molecular method in NP swab and stool samples and tried to assess their etiological role in children under 6 years of age that were hospitalized for AB, AGE, or FS. For the appraisal of the causal relationship we: i) included a control group and compared the frequency of HCoVs in patients with their occurrence in healthy children; ii) searched for the presence of several other viruses that might also be the cause of the illness, and iii) determined the estimated viral load of HCoV nucleic acids present (measured as Ct value), based on the idea that a high viral load (low Ct value) more reliably suggests a causative role than a low viral load (high Ct value).

The presence of HCoVs was established upon admission to the hospital in 58/718 (8.1%, 95% CI: 6.2–10.3%) NP swabs. The proportion of positive results was the highest in children with FS (9.9%), followed by AGE (8.7%) and AB (6.5%), but the differences between the three groups were not statistically significant. HCoVs were more often detected in NP swabs of patients than in healthy controls (8.1% versus 1.9%, OR = 4.5, 95% CI: 1.4 to 14.5, P = 0.01). The same was valid also for all three clinical groups (FS, AGE, AB). Although these findings support the causal role of HCoVs in our patients, this role remained inconclusive due to frequent co-detection of other viruses. Namely, in 36 (62.1%) of the 58 HCoVs-positive NP samples other respiratory viruses were detected, most commonly RSV (22/36, 61.1%). Within HCoVs NP swab-positive patients those with AB had the largest probability of being “co-infected” with other viruses (19/20, 95%), followed by FS (10/19, 52.6%) and AGE (7/19, 36.8%). The fraction of “co-infections” established in our patients with FS and AGE is similar to that reported previously [12, 15–17, 33, 34], whereas the proportion in children with AB is higher than in most other studies [35–39]. NP swab HCoVs estimated viral load was the highest in patients with FS (median Ct value 27.8), followed by AGE (median Ct value 29.5) and AB (median Ct value 33.5). Viral load in single HCoV “infections” was higher than in “co-infections” (median Ct values 27.5 versus 32, respectively; P = 0.09). This finding is in contrast to previous studies, in which no difference in viral load in single HCoV “infections” and “co-infections” was found [34].

Our findings suggest that in children with AB the presence of HCoVs in NP swabs is of minor importance and is most probably not causally related to AB because, among the three clinical groups, patients with AB had the lowest proportion of HCoV-positive NP swab results, very frequent “co-infection” with other respiratory viruses (95%), and very low viral load.

Interpretation of the role of HCoVs in AGE is complex because the viruses were more often demonstrated in NP swabs than in stool samples (it has been suggested that HCoVs detected in stools may be there because of swallowing)[12, 20, 40], because more than half of the children with AGE had also signs and symptoms of respiratory infection, because viral load was low and was higher in NP swabs than in stool samples, and because more than one-third of patients also had other viruses demonstrated in NP swabs. These findings suggest that HCoVs play only a minor role in gastrointestinal illness in children under 6 years old and go in parallel with the results of a Finnish study, in which HCoVs were more commonly detected in NP swabs than in stool samples in children with AGE, children with respiratory infection, and the group with combined gastrointestinal and respiratory symptoms [40].

It seems that among three clinical syndromes an etiological role of HCoVs is most likely in children with FS: in this group of patients the proportion of positive HCoV NP swab results was higher than in patients with AB and those with AGE, and it was significantly higher than in control children. Patients with FS had the highest viral load, but the co-detection of other viruses was 52.6%. HCoVs are molecularly related in structure and mode of replication with neuroinvasive animal coronaviruses [41], the relatedness does also attest for neuroinvasiveness of HCoVs [42]. Furthermore, since the pathogenesis of FS is not (primarily) based on neuroinvasiveness, this potential feature would not directly explain the association with FS.

At follow-up testing (14-days after the initial sampling), when 67.8% (364/537) of children were symptom-free, the proportion of HCoVs-positive results compared to baseline was smaller (35/537, 6.5% versus 58/718, 8.1%) but the difference was not statistically significant. Patients who were HCoVs-positive at the initial sampling were 5.5 times more likely to be HCoVs-positive at follow-up examination, and all first and follow-up detected viruses in individual patients were of congruent types. However, only 10/35 (28.6%) samples were positive on both occasions; in this subgroup the viral load was lower at follow-up than at initial testing (Fig 2). The increase in viral load was observed in only two samples; in both NL63 was demonstrated. At present we do not have a reliable explanation for the surprising finding that 25/35 (71.4%) samples were positive only at follow-up testing but not at the time of acute illness and that the occurrence of the positive results at follow-up testing (25/493, 5.1%) was higher than the occurrence 3/156 (1.9%), found in the control group (p = 0.043). This finding additionally complicates interpretation of the etiologic role of HCoVs.

Previous studies have reported on the seasonal appearance of HCoVs and their more pronounced occurrence every three to four years [12, 43–45]. A strong association with seasonality was also found in this study (P<0.001); the majority of HCoVs was detected in February and March both years (Fig 1).

Of 58 HCoVs detected in NP swabs obtained upon admission from patients with infections, 23 (39.6%) belonged to OC43, 19 (32.7%) to HKU1, 8 to NL63 (13.8%), and 5 (8.6%) to 229E, but 3 (5.2%) remained untyped because of their low viral load. All four HCoVs species were detected in each of the clinical manifestations; however, HKU1 was the most common HCoVs in children with AB (9/20, 45%) and AGE (7/19, 36.8%), whereas in children with FS OC43 was detected most frequently (12/19, 63.1%) (Table 2). These findings are in contrast to previous reports in which a lower detection rate of HCoVs and a predominance of HKU1 in children with FS was found [12, 34, 46]. Analyzing published information on other syndromes revealed that in the group of children with combined gastrointestinal and respiratory symptoms all four HCoVs were detected, whereas in those with solely gastrointestinal symptoms OC43, HKU1, and NL63 but not 229E were demonstrated [40]; that in children with acute respiratory tract infections (lower and upper) all four HCoVs species were established and that the predominance of species varies with country, study period, and study population [4, 34, 36, 47, 48]; and that in children with AB information on HCoVs species is rather limited [39, 49, 50].

Amplification and sequencing of genes representative for *Alphacoronavirus* and *Betacoronavirus* was performed in a subgroup of 12 HCoVs; in as many as 5/12 (41.7%) the sequences were unique.

## Conclusion

In conclusion, all four HCoVs were detected in children under 6 years old with AB, AGE, or FS. The presence of HCoVs and more frequent demonstration of these viruses in patients than in the control group of healthy children point toward an etiologic role of HCoVs. However, interpretation is complex. The causative role of HCoVs is most probably minor in children with AB and in patients with AGE but is more likely in children with FS, considering that they had a higher proportion of positive HCoVs results than patients with AB and those with AGE, and had the highest viral load; however, in half of the patients the co-detection of other viruses was established.

## Supporting Information

S1 Appendix Ethics Approval. (PDF)
