# Supplementary material for: The Role of Human Coronaviruses in Children Hospitalized for Acute Bronchiolitis, Acute Gastroenteritis, and Febrile Seizures: A 2-Year Prospective Study
Source: PLoS One. 2016 May 12;11(5):e0155555. doi: 10.1371/journal.pone.0155555 (PMC4865086; doi:10.1371/journal.pone.0155555)
Supplement: S1 Appendix — (PDF) [file pone.0155555.s001.pdf]

SLOVENIA: Ethical aspects

Since the 1960s there has been a formal system for ethical review of medical research in Slovenia. Although there is no specific law regulating biomedical research on humans in Slovenia, the Oviedo Convention (*Convention for the Protection of Human Rights and Dignity of the Human Being with Regard to the Application of Biology and Medicine: Convention on Human Rights and Biomedicine. Council of Europe (Directorate of Legal Affairs). European Treaty Series - No. 164. Oviedo, 4 November 1997: 1-11;* and *Explanatory Report to the Convention for the Protection of Human Rights and Dignity of the Human Being with Regard to the Application of Biology and Medicine: Convention on Human Rights and Biomedicine. Council of Europe (Directorate of Legal Affairs), DIR/JUR 5. Strasbourg, 1997: 1-35.*) has been in force since 1 December 1999. This important legally binding international instrument contains rather detailed provisions on biomedical research. Slovenia's delegate to the Steering Committee on Bioethics in the Council of Europe is a member of the Working Party drafting the Additional Protocol to the Convention, on biomedical research (*Draft additional Protocol to the Convention on Human Rights and Biomedicine, on Biomedical Research. Steering Committee on Bioethics (CDBI), Strasbourg 2002(12), 1-14*), and the principles of the draft protocol have been a basis for the national guideline to prospective researchers, published in 1998 (*Ethics of biomedical research on human beings. Guidelines to drafters of research protocols and applicants for ethical review*).

At present Slovenia has no specific law regulating biomedical research on human subjects. However, provisions related thereto can be found in several laws, such as:

• Law on Medical Practice (Ur.l. RS, No.:98/99 and 67/02.), where according to Art. 47 every medical intervention must be covered by an informed consent of the patient or, in case of a child or incompetent adult, the patient's parents or guardians.

• Penal Code (Ur.l. RS, No.: 63/94, 70/94 and 23/99), where, according to Art. 190, every negligent medical malpractice resulting in the serious damage to a person's health is punishable with up to one-year's imprisonment.

• The Slovenian Code of Medical Deontology contains, in Articles 47-50, provisions on ethical conduct of biomedical research on human subjects.

• The Helsinki Declaration of the World Medical Association continues to provide an essential guidance for researchers, drafters of protocols of clinical studies, and for ethical review, and has in Slovenia a de facto status of obligatory guideline.

Thus, the current practice in assuring observance of ethical principles in biomedical research to a large extent relies on the **Helsinki Declaration** (*World Medical Association, Declaration of Helsinki: Recommendations Guiding Physicians in Biomedical Research Involving Human Subjects. Adopted by the 18th World Medical Assembly, Helsinki, Finland, June 1964*), on the **Oviedo Convention** and the provisions of the **Draft Protocol to the Oviedo Convention, on biomedical research**. The National Medical Ethics Committee (NMEC), which reviews most of the biomedical research on human subjects, is the main body responsible for ethics of biomedical research in Slovenia. The ethical committees within the regional hospitals are authorized to review local studies not presenting any serious risk or burden, for example, non-invasive and observational research. Ethical review is required for all research involving intervention on, or interaction with human beings. Additionally, it is required for all research on personal medical data, and biological material of human origin.

All research involving intervention on or interaction with living persons has to be reviewed and approved before the actual work may be started. This also applies to research on biological materials of human origin, i.e., tissue and blood samples, both prospectively collected and archived, even if anonymous. Ethical review is also obligatory for research on personal medical data, including epidemiological studies. Sociological and psychological studies based on questionnaires, which invade privacy with potential harmful effects for persons involved, are also subject to ethical review.

It is common practice of the NMEC to require the consent in all cases of direct intervention on, or interaction with, persons concerned. In principle, informed consent is also required for research on archived personal medical data or biological samples. This requirement may be waived by the NMEC if seeking consent would involve unreasonable efforts and in as far as the risk of undue invasion of privacy is minimal. In some cases of so-called observational research, where data is rendered anonymous and where there is no possibility of violation of human rights, in particular the right to private life, informed consent is not needed. It is, however, for the research ethics committee to decide whether the duty of obtaining consent is there or not.

Consent by the person concerned may not be sufficient when he/she is legally or de facto incompetent or unable to take decisions in his/her best interest. Such is the case with children and adolescents below the age of legal competence or persons with a mental disorder.

Children are considered legally competent beyond the age of 15 (Law on health services, Art. 47/II). For younger children where authorisation is sought from their legal representatives, the NMEC requires that the child takes part in the decision process (Article 17 of the Draft Research Protocol). Research on a child who objects to the procedures proposed may not be started nor continued. An adult not able to consent, e.g. because of a mental disorder, must be allowed to take part in the authorisation procedure as far as possible (Article 17 of the Draft Research Protocol).

No specific national regulations exist in regard research involving human biological material, except for the ones related to blood transfusion and organ transplantation. Never the less, the NMEC decided that informed consent is required for such research. However, the donors of the biological material were not considered to have a right to financial benefit (apart from reimbursement for actual expenses incurred by them), in accordance with Article 21 of the Oviedo Convention (the human body and its parts shall, as such, not be a source of financial gain).

Personal data collection is covered by Protection Act (Ur.l. RS, No.:59/99, 57/01,59/01 and 52/02.). It includes any data that may reveal properties, capacity, state or relationships of a person, regardless of the form of those data. According to Art. 38 of the Constitution of the Republic of Slovenia (Ur.l. RS, No.:33/91 and 42/97.), the protection of personal data shall be guaranteed. Personal Data protection Act forbids any use of personal data in disagreement with the original purpose for which they were collected. In the ethical review of biomedical research on human beings, particular attention is paid to assuring adequate protection of personal medical data, as well as other personal data.

Application to the National Medical Ethics Committee

SUMMARY OF THE COMMITTEE OBJECTIVES, KEY MEMMBERS AND APPLICATION PROCES:

The National Medical Ethics Committee of the Republic of Slovenia (NMEC) operates in compliance with the ICH/GCP requirements of good clinical practice and all the applicable regulations of the Republic of Slovenia.

The Chairman of the NEMEC is prof. dr. Božidar Voljč, DrSc, MD, Ministry of Health, Štefanova 5, 1000 Ljubljana, phone: + 386 1 5438241, e-mail: bozidar.voljc@.uni.lj.si. All the information about NEMEC can be obtained at: http://www.kme-nmec.si/

Application for Ethical Review of Proposed Research Studies Involving Intervention on, or Interaction with, Human Subjects.

National Medical Ethics Committee (NMEC) requires that the following information / documents (in a single copy) is submitted (as applicable):

1. The full title of the research study, including its code.

2. The name and short professional CV of the principal / responsible investigator; the institution proposing the research and the one where the research will be conducted.

3. The name and professional qualifications of the doctor responsible for safety of human subjects in the study.

4. The design and the complete protocol of the study, including the

 aim and scientific rationale, supported by a review of the recent literature

 methods, including statistics

the outcome of the peer review (if available)

 the proposer's own perception of ethical issues involved.

 a summary / synopsis in Slovene lay language (about 1 page)

5. Information on the subjects/patients invited to participate:

 methods of recruitment

 a statement by the investigator that recruitment will not be associated with improper pressure or inducement, financial or other

 inclusion, non-inclusion, exclusion criteria, anticipated number

 any financial compensation or other benefit offered to participants.

6. Safety arrangements

 the nature and likelihood of the risks and burdens envisaged

 arrangements in place for handling any adverse events

- in case of possible mutagenic or teratogenic effects, what kind of protective measures will be undertaken.

7. Where control group is envisaged, how will their interests and right to proper medical care be assured.

8. Arrangements for confidentiality of personal data and the right to privacy.

9. Will the participants have access to results collected on their health and to the general outcome of the study?

10. Information on the sponsor of the study: name, address, responsible coordinator. Same for the CRO, where applicable. Additionally:

- insurance policy (coverage for the necessary treatment in case of damage to health, compensation for the case of injury / disability
- any financial award to the investigators and the supporting team.

11. Any previous ethical review by the same or other ethics committee for the same or related research.

12. Statement by the Head of the institution or department where the study is to be conducted that he / she agrees to the study being conducted within the premises / under responsibility of the institution, that the responsible researchers are capable of recognizing dangerous adverse events and of taking appropriate care of any clinical contingency. A statement that they will adhere to the principles of the Helsinki Declaration, the Oviedo Convention on Human Rights and Biomedicine and the Slovene Code of Medical Deontology.

13. Statement of the responsible investigator that he / she would adhere to the principles of the Helsinki Declaration, the Oviedo Convention on Human Rights and Biomedicine and the Slovene Code of Medical Deontology. Declaration of the existing or potential conflict of interests.

14. Information for the participants or their legal representatives (in case of persons unable to consent). Risks, burdens and benefits must be properly explained in Slovene lay language. Informed Consent form. The subject or patient must be informed that he / she has a right to refuse or later on at any time withdraw the consent without suffering any detriment. If biological samples will be collected and part of the material is to be stored for possible future

uses, a separate consent to this is required. The patient should usually be offered different options: not to agree to storage, to agree only to certain types of future research (e.g. excluding genetic studies), to be asked for consent to any future studies, or to give a blanket consent to all future uses. When giving authorisation for future studies, this should be with the understanding and explicit obligation that a prior approval by an independent ethics committee will be obtained.

15. Details about how the subjects / patients can contact the responsible doctor in case of emergency.

16. In research on patients in fertile period where risk exists of mutagenicity, special information on the risks and a statement by the patient / subject that appropriate measures will be taken to prevent pregnancy.

17. Date of application and signature of the proposer.

18. After approval had been issued, the Principal Investigator, the Sponsor or the CRO have a duty to inform the Committee on any changes in the protocol that may affect the ethical aspects of the study. Premature termination must be promptly reported and reasons must be given. Upon conclusion of the study, a final report on the outcomes must be supplied, even in case of a negative result.

19. Safety reports - until further notice

- *Suspected unexpected serious adverse events/reactions* (SUSARs) in multinational trials recorded in the national centres must be expeditely reported.
- Prompt reporting to the NMEC of SUSARs by the sponsor/CRO or the responsible investigator is also required when there are reasons to suspect that risk to the participants could exceed the anticipated or acceptable level, or that the originally estimated risk/benefit ratio is unfavourably changed. This includes cases of unexpectedly frequent occurrence of the anticipated *severe adverse events* (SAEs). Regarding other SAEs, quarterly, semiannual or annual safety reports are sufficient.
- Reports on SUSARs suggestive of an increased level of risk to the participants must be accompanied with a comment by the sponsor/CRO or the clinically responsible principal investigator. The same applies to annual safety reports.

 The reports can be sent by e-mail to tone.zakelj@kclj.si as PDF files attached to the main message, accompanied by data needed to identify the study (e.g., reference to earlier correspondence with the NMEC).

20. In all further correspondence on the same study, quote the NMEC reference No. of the latest correspondence.

21. The above documents may be supplied in English or in Slovene language. However, items 4 (last line), 14, 15, and 16 must be in Slovene. According to the rules, the NMEC will respond in Slovene. In certain justified cases and as an exception, the opinion may be given in English

22. The NMEC holds meetings once every month, including the summer months. To be put on the agenda, applications must be received by regular mail (only original, no copies needed; e-submission unavailable) at least one week prior to the session (address: The National Medical Ethics Committee, Dr. Božidar Voljč, MD, PhD, Chairman, Ministry of Health, Štefanova 5, SI-1000 Ljubljana, Slovenia). A typical response time is less than 4 weeks. As an exception, preliminary opinion may be issued 'by chairman's action' in order to gain time for other official procedures.

Personal data collection is covered by Protection Act (Ur.l. RS, No.:59/99, 57/01,59/01 and 52/02.). It includes any data that may reveal properties, capacity, state or relationships of a person, regardless of the form of those data. According to Art. 38 of the Constitution of the Republic of Slovenia (Ur.l. RS, No.:33/91 and 42/97.), the protection of personal data shall be guaranteed. Personal Data protection Act forbids any use of personal data in disagreement with the original purpose for which they were collected. In the ethical review of biomedical research on human beings, particular attention is paid to assuring adequate protection of personal medical data, as well as other personal data.

Application to the National Medical Ethics Committee (in Slovenian language)

mag. Marko Pokorn, dr. med.
Klinika za infekcijske bolezni in vročinska stanja
Japljeva 2, 1525 Ljubljana

Komisija Republike Slovenije za medicinsko etiko
Inštitut za klinično nevrofiziologijo
Zaloška 7, 1525 Ljubljana

Zadeva: Prošnja za oceno etične sprejemljivosti načrta raziskave z naslovom »Povzročitelji in klinične slike virusnih okužb dihal in prebavil pri otrocih, mlajših od 6 let«

Spoštovani,

v imenu nosilcev raziskave prosim za oceno in mnenje o etični neoporečnosti raziskave z naslovom **»Povzročitelji in klinične slike virusnih okužb dihal in prebavil pri otrocih, mlajših od 6 let«**.

Uvod: Virusne okužbe dihal in prebavil so najpogostejši vzrok za obolevanje in bolnišnično zdravljenje predšolskih otrok. Med povzročitelji okužb dihal je na prvem mestu respiratorni sincicijski virus, med povzročitelji okužb prebavil pa rotavirus. V zadnjih letih se je z metodo verižne reakcije s polimerazo (VRP) mikrobiološka diagnostika omenjenih okužb zelo izboljšala, odkritih je bilo sedem novih virusov iz petih družin. Ker nove metode v vzorcih dokazujejo le genetski zapis virusov, vloge omenjenih virusov v samem bolezenskem dogajanju ne pojasnjujejo. Raziskav, ki bi primerjale pogostnost prisotnosti virusov v vzorcih dihal in prebavil pri otrocih z okužbami dihal in prebavil z zdravimi otroki, je malo. Malo je tudi raziskav, ki bi spremljale specifični imunski odgovor na omenjene viruse pri obolelih otrocih. Tako vloga novo odkritih virusov pri okužbah dihal in prebavil pri otrocih ni zadovoljivo pojasnjena.

Namen: Namen raziskave je ugotoviti, kako pogosto pri otrocih z okužbami dihal in prebavil v zgornjih dihalih dokažemo virusno DNK in viruse v vzorcih blata ter dobljene rezultate primerjati s kontrolno skupino zdravih otrok. Prav tako nas zanima, kako pogosto bomo v omenjenih vzorcih dokazali viruse pri otrocih z vročinskimi krči. Poleg tega bomo iskali specifični imunski odziv na viruse pri obolelih otrocih ter tako poskusili dokazati, da je res šlo za okužbo.

Trajanje, mesto raziskave: Raziskava bo trajala leto dni. Vanjo bodo vključeni otroci, mlajši od 6 let (do 6. rojstnega dne), ki bodo zaradi akutnega bronhiolitisa ali druge okužbe dihal ali okužbe prebavil sprejeti na Kliniko za infekcijske bolezni in vročinska stanja. Poleg tega bomo v raziskavo vključili tudi vse otroke z vročinskimi krči, ki bodo sprejeti na kliniko.
Kontrolno skupino bodo predstavljali otroci, mlajši od 6 let, ki bodo zaradi elektivnega kirurškega posega sprejeti na Klinični oddelek za otroško kirurgijo in intenzivno terapijo Univerzitetnega kliničnega centra v Ljubljani.

Obremenitev za preiskovance: Bolnikom bomo dvakrat v razmaku 14 dni odvzeli bris nosnožrelnega prostora, blato ter vzorec venske krvi, kontrolni skupini pa le enkrat bris nosnožrelnega prostora in blato.

Metode dela: Prilagamo protokol. Protokol obravnave bolnikov se sicer ne razlikuje od siceršnje obravnave bolnikov z akutno okužbo dihal ali prebavil na naši kliniki, dodatno bomo pri bolnikih odvzeli bris nosnožrelnega prostora, blato in vzorec venske krvi še po 14 dneh.

Nosilci raziskave: mag. Marko Pokorn, dr. med., mag. Tatjana Mrvič, dr. med., doc. dr. Janez Primožič, dr. med., Monika Jevšnik, dipl. mikrobiol., dr. Mateja Poljšak Prijatelj, dipl. biol., asist. dr. Andrej Steyer, dipl. mikrobiol., doc. dr. Miroslav Petrovec, dr. med., prof. dr. Franc Strle, dr. med.

Pričakujemo, da bo raziskava pripomogla k boljšemu razumevanju vloge posameznih novih virusov pri okužbah dihal in prebavil pri otrocih.

Zahvaljujem se za Vašo obravnavo in Vas lepo pozdravljam.

mag. Marko Pokorn, dr. med.

Priloženi:

1. Protokol z metodami dela
2. Vprašalnik za bolnike
3. Obveščen pristanek za bolnike
4. Vprašalnik in obveščen pristanek za kontrolne osebe

Ljubljana, 24. julija , 2009

Application to the National Medical Ethics Committee (in English language)

Marko Pokorn, MD
Department of Infectious Diseases
University Medical Centre Ljubljana
Japljeva 2, SI-1525 Ljubljana
Slovenia

The National Medical Ethics Committee of the Republic of Slovenia
Institute of Clinical Neurophysiology
Zaloška 7, SI-1525 Ljubljana
Slovenia

TO WHOM IT MAY CONCERN

Application for assessment of ethical research entitled:

Viral respiratory and gastrointestinal infections in children under 6 years of age (VODP)

The aim of this study is to use molecular methods to detect viruses in upper respiratory tract and gastrointestinal tract of children with acute bronchiolitis, acute gastroenteritis and febrile convulsions and try to correlate the severity of clinical picture with the amount of viruses present in clinical specimens.

Children under 6 years of age with acute bronchiolitis, acute gastroenteritis and febrile convulsion that will be admitted in to the Department of Infectious Diseases, University Medical Centre Ljubljana will be included in to the study and healthy control children who will be admitted to the Department of Pediatric Surgery and Intensive Care for elective surgical procedures. All specimens will be sent to the laboratory of the Institute of Microbiology and Immunology, Faculty of Medicine, University of Ljubljana for the routine detection of respiratory and enteric viruses by molecular methods.

Best regards,

Marko Pokorn, MD

Ljubljana, 24th of July, 2009

Study protocol

Investigated groups

- Acute bronchiolitis (the presence of nasal discharge, cough, wheezing and/or crackles on lung auscultation) AB
- Acute gastroenteritis (patients with 3 or more loose or liquid stools in 24 hours prior to entering study) AG
- Febrile convulsion (patients with a cerebral paroxysm accompanied by fever without signs of central nervous system infection) VK
- Healthy control group (children without respiratory and/or gastrointestinal tract infection admitted for elective surgical procedures)

Inclusion criteria

- Children up to 5 years
- Presence of acute bronchiolitis and/or acute gastroenteritis and/or febrile convulsion requiring hospital admission

Patients

Monitoring the course of disease in patients (clinical and laboratory). Data obtained at enrollment in the study, during hospitalization and at follow-up after 14 days.

At enrolment

- anamnestic data and clinical examination
- nasopharyngeal swab (detection of respiratory viruses)
- stool samples (detection of gastroenteritic viruses)
- whole blood (1 ml) for storage

At follow up after 14 days

- anamnestic data and clinical examination
- nasopharyngeal swab for storage
- stool samples for storage
- whole blood (1 ml) for storage

Healthy control group

- anamnestic data and clinical examination at enrollment
- nasopharyngeal swab for storage
- stool samples for storage

Obveščen pristanek za bolnike za raziskavo »Povzročitelji in klinične slike virusnih okužb dihal in prebavil pri otrocih, mlajših od 6 let«

Ime otroka_____________________________, Datum rojstva_________________________________

Naslov otroka__________________________

Ime in priimek lečečega zdravnika___________________________________

Telefonska številka lečečega zdravnika____ ___________________________

Ime in priimek vodij raziskave: mag. Marko Pokorn, dr.med in Tatjana Mrvič, dr.med

Telefonska številka vodij raziskave: 041712241 in 041787734

Otroku bomo ob vključitvi v raziskavo odvzeli:

- **bris nosnega dela žrela**
- **vzorec blata**
- **vzorec krvi (EDTA) 1 ml**

Na kontrolnem pregledu po 14 dneh bomo otroku odvzeli:

- **bris nosnega dela žrela**
- **vzorec blata**
- **vzorec krvi (EDTA) 1 ml**

Starši otroka so bili natančno podučeni o vsebini in namenu raziskave »Povzročitelji in klinične slike virusnih okužb dihal in prebavil pri otrocih, mlajših od 6 let« dne: ____________________________

Podpisani ____, ki sem oče/mati

____, sem seznanjen s potekom

raziskave in soglašam, da mojega otroka vključite vanjo.

Kraj in datum:__________________________

Podpis: ________________________________

Inform consent for participation in the study »Viral respiratory and gastrointestinal infections in children under 6 years of age«

Full name of the child (first and last)________________________, Date of birth______________________

Address_________________________

Full name (first and last) of attending physician __________________________________

Phone number of attending physician______________________________

Full name of Principal Investigator: Marko Pokorn, MD and Tatjana Mrvič, MD

Phone number of Principal Investigator: 00386 41712241 in 00386 41787734

At child enrolment we will take:

- **nasopharyngeal swab**
- **stool samples**
- **whole blood (1 ml)**

At follow up after 14 days we will take:

- **nasopharyngeal swab**
- **stool samples**
- **whole blood (1 ml)**

Children parents understand and agree with everything explained above and therefore ratify it with signature.

Signed____, I am father/mother .

Place and date:__________________________

Signature: _______________________________

Approval of the purposed study by the National Medical Ethics Committee

The National Medical Ethics Committee of the Republic of Slovenia has approved the above mentioned study on the 16th September 2009 (reference number 87/08/09).

The National Medical Ethics Committee is also the Governmental Medical Ethics Committee, therefore their approval of the study is also the Governmental Approval.

Approval of the purposed study by the National Medical Ethics Committee (in Slovenian language)

KOMISIJA REPUBLIKE SLOVENIJE ZA MEDICINSKO ETIKO

Mag. Marko Pokorn, dr. med.
Klinika za infekcijske bolezni in vročinska stanja
Univerzitetni klinični center Ljubljana
Japljeva 2, 1525 Ljubljana

Štev.: 87/08/09
Datum: 16. 9. 2009

Spoštovani gospod mag. Pokorn,

Komisiji za medicinsko etiko (KME) ste 24. 7. 2009 poslali prošnjo za oceno načrta raziskave z naslovom:

"Povzročitelji in klinične slike virusnih okužb dihal in prebavil pri otrocih, mlajših od 6 let."

KME je raziskavo ocenila kot etično sprejemljivo in Vam s tem izdaja svoje soglasje.

S spoštovanjem in lepimi pozdravi,

prof. dr. Jože Trontelj
predsednik Komisije za medicinsko etiko

Naslov: Prof. dr. Jože Trontelj, Inštitut za klinično nevrofiziologijo, Klinični center Ljubljana
Zaloška 7, 1525 Ljubljana. Telefon 01/ 522 1500, telefax 01/ 522 1533, naslov za elektronsko pošto: joze.trontelj@kclj.si
Spletni naslov: http://www. kme-nmec.si/
